# Silicone tags as an effective method of monitoring environmental contaminant exposures in a geographically diverse sample of dogs from the Dog Aging Project

**DOI:** 10.3389/fvets.2024.1394061

**Published:** 2024-08-16

**Authors:** Rylee Matheson, Courtney L. Sexton, Catherine F. Wise, Janice O’Brien, Amber J. Keyser, Mandy Kauffman, Matthew D. Dunbar, DAP Consortium, Joshua M. Akey, Heather M. Stapleton, Audrey Ruple

**Affiliations:** ^1^Population Health Sciences Department, Virginia-Maryland College of Veterinary Medicine, Virginia Tech, Blacksburg, VA, United States; ^2^Nicholas School of the Environment, Duke University, Durham, NC, United States; ^3^Center for Studies in Demography and Ecology, University of Washington, Seattle, WA, United States

**Keywords:** exposure assessment, silicone wristbands, biomonitoring, passive sampling device, dog

## Abstract

**Introduction:**

Companion animals offer a unique opportunity to investigate risk factors and exposures in our shared environment. Passive sampling techniques have proven effective in capturing environmental exposures in dogs and humans.

**Methods:**

In a pilot study, we deployed silicone monitoring devices (tags) on the collars of a sample of 15 dogs from the Dog Aging Project Pack cohort for a period of 120 h (5 days). We extracted and analyzed the tags via gas chromatography–mass spectrometry for 119 chemical compounds in and around participants’ homes.

**Results:**

Analytes belonging to the following chemical classes were detected: brominated flame retardants (BFRs), organophosphate esters (OPEs), polycyclic aromatic hydrocarbons (PAHs), polychlorinated biphenyls (PCBs), pesticides, phthalates, and personal care products. The types and amounts of analytes detected varied substantially among participants.

**Discussion:**

Data from this pilot study indicate that silicone dog tags are an effective means to detect and measure chemical exposure in and around pet dogs’ households. Having created a sound methodological infrastructure, we will deploy tags to a geographically diverse and larger sample size of Dog Aging Project participants with a goal of further assessing geographic variation in exposures.

## 1 Introduction

People and pets are exposed to hundreds of potentially harmful and/or toxic chemicals every day (1, 2). Dozens of environmental contaminants representing several classes of compounds regularly found in and around the home and immediate outdoor environments have been associated with risks for chronic and adverse health outcomes (3–5).

Exposure to eight commonly-found classes of chemicals including pesticides, brominated flame retardants (BFRs), polycyclic aromatic hydrocarbons (PAHs), polychlorinated biphenyls (PCBs), personal care products, alkylphenols, organophosphate esters, and phthalates has been linked to health effects in many systems. Some such effects include endocrine and reproductive systems mutation and disruption, liver dysfunction and toxicity, respiratory and cardiac disease, neurotoxicity and neurodevelopmental disorders, among other health complications in humans (6–11). Many of these diseases are physiologically similar in dogs (12). Several studies have also demonstrated links between environmental exposures and companion animal health, a rapidly growing area of research (13–18).

Over the last 75 years, there has been a rise in production and distribution of plastics, batteries, chemical treatments for textiles (eg. stain and water repellents), commercially available pesticides, and other everyday products. This increase in use has caused an exponential rise in the number of chemicals we are exposed to via inhalation, ingestion, and transdermal exposure pathways (19–22). Several regulatory bodies (e.g., FDA, EPA, USDA, CDC, WHO) have set limits and, in some cases, bans, on production and usage of many of these compounds (23–27). Avoiding exposure as a protective measure is not always possible because these chemicals have long half-lives in the environment, which makes them persistent pollutants. Additionally, even when phased-out, people may keep products treated with them for decades resulting in continued exposure (28). Despite the ubiquity of and known health risks associated with chemical exposures, linking adverse health effects to chemicals by which exposure primarily occurs via inhalation or dermal absorption indoors can be especially difficult to track and measure in human populations (1).

Companion animals present an under-realized opportunity to better understand pathways of exposure to such harmful environmental contaminants in and around the home. Pet dogs and cats closely share both physical and social environments with humans. Moreover, the factors that degrade physiological processes are likely to have more immediate and potentially more drastic effects in pets than their longer-lived human companions with whom they share these environments (29, 30). And, while dogs’ lifespans are substantially shorter than our own (31, 32), they do live long enough to experience effects of harmful exposures over time. Thus, viewing dogs as sentinels of human health and wellbeing can enable us to more rapidly assess how environmental risk factors might cause illness in pets and people alike (30, 33).

The Dog Aging Project (DAP) population is a valuable resource for studying environmental exposures and health outcomes due to the responsive and interactive nature of this community and the frequency at which data are collected. Annual health and environmental data are collected throughout the lifespan of all participants enrolled in the DAP. Adding a quantitative component such as silicone passive samplers for detecting environmental chemical contaminants would allow for a more in-depth analysis of exposure related health outcomes.

In capturing exposure to BFRs, OPEs, PCBs, phthalates and pesticides, some of which can have both acute and chronic health impacts, Wise et al. (1, 5) described the use of silicone passive sampling (wearable tags) in 30 pairs of pet dogs and their human companions. Passive sampling strategies enable accessible, broad-scale, and diverse data collection. Here our aim was to test logistic and methodological infrastructure within the Dog Aging Project for future studies that will build and expand on this technique. Our goal was to employ passive sampling to evaluate various classes of chemical contaminants that dogs and humans may be exposed to on a daily basis. We deployed silicone monitoring devices (tags) to a sample of 15 dog participants in the Dog Aging Project for a pilot study.

## 2 Methods

### 2.1 Ethical considerations

The University of Washington IRB deemed that recruitment of dogs through their owners for the Dog Aging Project is research that qualifies for Category 2 human subjects exempt status (IRB ID no. 5988, effective 30 October 2018). All study-related procedures involving privately owned dogs were approved by the Texas A&M University IACUC, under AUP 2021-0316 CAM (effective 14 December 2021).

### 2.2 Recruitment

Dogs were enrolled from the Dog Aging Project (DAP), a long term, longitudinal study of aging and age-related health developments in companion dogs of dogs located throughout the United States (34). Dog owners are recruited through various media outlets and word of mouth to voluntarily nominate their dogs for participation. Owners who opt to enroll their dogs in the study are first led through an extensive informed consent process. Once consent is confirmed, owners are requested to complete the Health and Life Experiences Survey (HLES), which includes several small surveys that collect information about dog demographic characteristics, physical activity, environment, dog behavior, diet, medications and preventatives, dog health status and history, and owner demographic characteristics. Once the HLES has been completed, dogs are enrolled in the DAP Pack. An HLES Annual Follow-Up Survey (AFUS) is sent each year following enrollment. There are additional informed consent processes for dogs selected to participate in additional sampled cohorts, which may involve collecting such data as electronic veterinary medical records, genome-wide sequence information, clinicopathology and molecular phenotypes derived from blood cells, plasma, and fecal samples. The project began enrollment in 2019 and has over 47,000 dogs enrolled as of 1 January 2023. As an open data project, DAP data are available to the public at dogagingproject.org/open_data_access (2023). Any dog enrolled in the DAP whose owner had completed the baseline Health and Life Experience Survey (HLES) about their dog was eligible for participation in this pilot study.

For this pilot study, a call for participants was posted to the DAP “Dog Park” community, an online forum for DAP participants, using a Google Forms survey that remained open until 15 responses were received (see Supplementary material). The pilot participation survey collected the dog owner’s name, contact information, home address and the name of their dog enrolled in DAP. These owners were invited to attend an informational session via Zoom, during which we reviewed the purpose of the study, requirements for participation, items that would be included in the sampling kits, and instructions for attaching and removing the silicone tag, and answered participants’ questions. In order to participate, dogs had to be in their normal home environment experiencing their normal routine and had to wear a collar or harness in order to attach the silicone tag for the duration of the five-day sampling period.

### 2.3 Sample collection

Silicone tag monitoring devices were pre-cleaned, wrapped in aluminum foil and sealed in an airtight bag as described previously (1). Participants received a package containing the bag with the silicone tag and a fresh piece of aluminum foil, written instructions, an exposure survey (see Supplementary material), and a prepaid return mailer. Individual dogs wore tags for five consecutive days (120-h period) in their typical daily environment. Owners could select any 120-h window between the designated dates of March 1–15, 2023. Upon removal after the five-day period, owners were instructed to wrap the tags in the fresh piece of aluminum foil, place it into the airtight bag, and mail via prepaid envelope back to the lab. The time and date of application and removal were recorded on the exposure survey. Upon confirming receipt of their sample back to the lab, we invited owners to complete a feedback survey (see Supplementary material) regarding their experience with the process.

Along with the tags worn by dogs, we deployed three field blanks (contamination controls) that remained in the laboratory and were subsequently processed with the dogs’ samples.

### 2.4 Exposure survey

All owners completed an environmental exposure survey (see Supplementary material) during the sampling period. The goal of this survey was to collect data on potential sources of chemical exposure in the dog’s home environment and included questions about the following information: the age and type of home the dog lives in, how long the dog has lived in the home, how many hours per day the dog spends in the home, whether the dog is groomed professionally or at home and the frequency, exposure to smoke from both tobacco and non-tobacco sources, current medications, flea/tick prevention and frequency of use, heartworm prevention and frequency of use, herbicide/pesticide use in the home and yard, and the dog’s access to these areas following treatment.

Owner reported data regarding the household environment were summarized and descriptively analyzed (see Table 1). Geographic regions across the U.S. were consistent with those as defined by the National Geographic Society (35).

**Table 1:** Household information and known environmental exposures as reported by dog owners.

Characteristic	(*n*)	%
**Age of house**		
1–20 years	4	27
>20 years	11	73
**Home area type**		
Urban	2	13
Suburban	10	67
Rural	3	20
**Shoes worn inside home**		
Yes	8	53
No	7	47
**Time dog has lived in home**		
<6 months	1	7
6–11 months	1	7
1–3 years	9	60
4–8 years	4	27
**Average time dog spends inside home per day** ^*^		
9–12 h	1	7
13–18 h	4	29
19–23 h	8	57
24 h	1	7
**Dog exposed to passive tobacco smoke**		
Yes	0	0
No	15	100
**Grooming**		
**Professionally groomed**		
2–6 times per year	3	20
7–11 times per year	1	7
Not groomed professionally	11	73
**Groomed at home**		
2–6 times per year	5	33
7–11 times per year	1	7
Monthly	2	13
Weekly	3	20
Not groomed at home	4	27
**Flea and tick prevention**		
Used within last 12 months	13	87
Not used within last 12 months	2	13

^*^One (1) owner did not respond to this question.

### 2.5 Analysis

All tags (*N* = 15) and field blanks (*N* = 3) were returned to the lab for processing. They were analyzed for a suite of target compounds using gas chromatography–mass spectrometry, as has been previously described (1). Tags were screened for a total of 119 analytes belonging to eight chemical classes including: brominated flame retardants, pesticides, phthalates, polychlorinated biphenyls, polycyclic aromatic hydrocarbons (PAHs), organophosphate esters, alkylphenols and personal care products (PCPs). Field blanks were used to assess contamination during sample shipment and processing, and to determine method detection limits (MDLs) for each analyte as previously described (1). MDLs are the minimum level of analyte concentration that can be detected during analysis. Detection frequency, median level, and range were calculated for all 119 compounds.

## 3 Results

All participants (*N* = 15) from 12 different states across the U.S. who opted into the study and received a sampling kit successfully implemented the at-home procedure and returned the used monitoring tags to the lab.

### 3.1 Sample group demographics

Dogs enrolled in the study included mixed breed and purebred dogs representing 17 different breeds in total (Table 2). Eight were regarded as large breed dogs (weight at time of enrollment ≥ 20 kg) and seven were regarded as small breed dogs (weight at time of enrollment < 20 kg). There were nine male and six female dogs in the study, and eight young adult and seven mature adult dogs, per the DAP HLES.

**Table 2:** Dog and owner demographic characteristics, Dog Aging Project silicone tag pilot study, 2023.

Human owner demographic characteristics	(*n*)	%	Dog demographic characteristics	(*n*)	%
**Age range**			**Breed status**		
18–24 years	1	7	Purebred	8	53
25–34 years	3	20	Mixed breed	7	47
45–54 years	1	7	**Sex/reproductive status**		
55–64 years	2	13	**Male**		
65–74 years	8	53	Neutered	8	53
**Education level**			Intact	1	7
Some college credit, no degree	2	13	**Female**		
Associate’s degree, trade, technical or vocational training	3	20	Spayed	6	40
Bachelor’s degree or higher	10	67	Intact	0	0
**Annual household income**			**Size**		
<$80,000	7	47	Small (<20 kg)	7	47
>80,000	4	27	Large (>20 kg)	8	53
Prefer not to answer	4	27	**Life stage**		
**Race**			Young adult	8	53
White			Mature adult	7	47
Non-Hispanic	13	87	**Household geographic region**	**(*n*)**	**%**
Hispanic	1	7	Northeast	3	20
American Indian	1	7	Southeast	4	27
			Midwest	2	13
			Southwest	2	13
			West	4	27

Four dog households were located in the U.S. West, two in the Midwest, two in the Southwest, three in the Northeast, and four in the Southeast (35).

Human dog owners who opted to enroll their dogs ranged in age from 18 to 75. All owners reported having attained an education level of at least some college, with a doctorate degree being the highest attained level. Reported annual income range was ≤$20,000 to ≥$180,000. Fourteen out of 15 human participants self-identified as White and one person self-identified as American Indian. Among the 15 participants, one person identified as being of Hispanic origin.

Sixty percent (9/15) of owners reported believing their dog to be in excellent health, and all but one (14/15) reported their dog in at least good health. Nearly half of dogs (7/15) included in this pilot study were reported to have a newly developed or diagnosed health condition (within the last year) at time of survey.

### 3.2 Reported household/environmental exposures

Participants responded to a questionnaire regarding their dog’s home environment (indoor and exterior) and routine behaviors in and around the home (Table 1). Two-thirds of participants (10/15) reported living in a suburban environment, two in a rural area, and three in urban areas.

Seventy-three percent of participants (11/15) lived in homes that were ≥20 years old. More than half of dog owners (8/15) reported wearing shoes in the home. No dogs are reported to have been exposed to passive tobacco smoke.

All but two dogs (13/15) had lived in the home for at least 1 year at the time of study. The majority of dogs (9/15) reportedly spent at least 19 h per day inside of the home. Four dogs had been professionally groomed at least twice annually, while an additional seven had received in-home bathing/grooming at various intervals throughout the year.

Eighty-seven percent (13/15) of the dogs had been treated with pest preventative (oral, topical, or collar) at least once in the 12 months leading up to time of study, and 87% had been treated with heartworm preventative at least once in the preceding year. One-third of dog owners (5/15) reported having used a chemical herbicide or pesticide treatment in or around the home at least once during the preceding year.

### 3.3 Chemical analyses

Using passive sampling monitoring devices (silicone tags) placed on dog’s collars, we detected analytes belonging to all of the chemical classes for which testing was performed. Results for each chemical, in each chemical class, are reported in Table 3, including their detection frequency, median level, and range. Details with full results for each individual tag can be seen in Supplementary Table S1.

**Table 3:** Median, range, and detection frequency for all chemicals tested in *n* = 15 samples.

Chemical class	Chemical name	Chemical name abbreviation	Median of samples (ng/g)	Sample ranges (max ng/g)	Detection frequency (*n* samples; %)
Organophosphate esters	2-Isopropylphenyl diphenyl phosphate	2IPPDPP	5.34	60.9	15; 100%
	3-Isopropylphenyl diphenyl phosphate	3IPPDPP	9.07	9.07	1; 7%
	2-tert-butylphenyl diphenyl phosphate	2tBPDPP	NA	NA	0
	4-Isopropylphenyl dipheynyl phosphate	4IPPDPP	NA	NA	0
	Bis(2-isopropylphenyl) phenyl phosphate	B2IPPPP	10.1	15.1	2; 13%
	2,4-Diisopropylphenyl diphenyl phosphate	24DIPPDPP	7.18	32.2	14; 93%
	4-tert-butylphenyl diphenyl phosphate	4tBPDPP	5.21	14.4	15; 100%
	Bis (3-isopropylphenyl) phenyl phosphate	B3IPPPP	NA	NA	0
	bis(2-tert-butylphenyl) phenyl phosphate	B2tBPPP	NA	NA	0
	Bis (4-isopopylphenyl) phenyl phosphate	B4IPPPP	0.37	1.19	3; 20%
	Tris(3-isopropylphenyl) phosphate	T3IPPP	NA	NA	0
	Bis (2,4-diisopropylphenyl) phenyl phosphate	B24DIPPPP	9.79	23.6	5; 33%
	bis(4-tert-butylphenyl) phenyl phosphate	B4tBPPP	6.66	7.68	3; 20%
	Tris(4-isopropylphenyl) phosphate	T4IPPP	NA	NA	0
	Tris(4-tert-butylphenyl) phosphate	T4tBPP	NA	NA	0
	Triethyl phosphate	TEP	11.1	68.9	13; 87%
	Triisopropyl phosphate	TiPP	NA	NA	0
	Tripropyl phosphate	TPrP	NA	NA	0
	Tri-iso-butyl-phosphate	TiBP	2.3	28.5	15; 100%
	Tri-n-butyl-phosphate	TnBP	13.4	42.5	8; 53%
	Tris (2-chloro-ethyl) phosphate	TCEP	4.95	29.7	8; 53%
	Tris (chloroisopropyl) phosphate	TCPP1	163	1,020	15; 100%
		TCPP2	24	141	15; 100%
		TCPP3	1.81	19.1	12; 80%
	Tripentyl phosphate	TPeP	NA	NA	0
	Tris (2,4-dichloro-isopropyl) phosphate	TDCPP	23.8	571	14; 93%
	Tri-(2-butoxyethyl)-phosphate	TBOEP	45.1	146	12; 80%
	Triphenyl phosphate	TPHP	22.1	109	15; 100%
	2-Ethylhexyl diphenyl phosphate	EHDPP	17.4	295	15; 100%
	Tris(2-ethylhexyl) phosphate	TEHP	6.77	33.4	15; 100%
	Isodecyl diphenyl phosphate	IsodecylPP	13.9	364	13; 87%
	Tri-o-cresyl phosphate	ToCP	9.2	16.6	10
	Tri-m-cresyl phosphate	TmCP	8.96	8.96	1; 7%
	Tri-p-cresyl phosphate	TpCP	NA	NA	0
	Tris(3,5-dimethyl phenyl) phosphate	TDMPP	NA	NA	0
Personal care product	Lilial		37.7	2070	15; 100%
Polycyclic aromatic hydrocarbons (PAHs)	3-Methylcholanthrene		NA	NA	0
	7,12-Dimethylbenz(a)anthracene		0.33	0.37	4
	Acenapthene		6.05	7.26	3; 20%
	Acenaphthylene		1.14	1.19	9; 60%
	Anthracene		1.87	12	14; 93%
	Benz[a]anthracene		1.79	9.21	9; 60%	
	Benzo[a]pyrene		0.6	2.62	15; 100%
	Benzo(c)phenanthrene		0.73	3.54	14; 93%
	Benzo[e]pyrene		1.01	7.54	15; 100%
	Benzo(g,h,i)perylene		0.17	3.43	14; 93%
	Benzo[j,b,k]fluoranthene		2.67	23.7	15; 100%
	Chrysene		1.28	33.4	13; 87%
	Dibenz(a,h)anthracene		0.11	0.11	2; 13%
	Dibenz[a,l]pyrene		0.12	0.13	2; 13%
	Dibenz[a,i]pyrene		NA	NA	0
	Dibenz[a,h]pyrene		0.12	0.12	2; 13%
	Fluoranthene		8.52	71.9	15; 100%
	Fluorene		2.75	5.36	13; 87%
	Indeno(1,2,3-cd)pyrene		0.21	3.75	14; 93%
	Naphthalene		NA	NA	0
	Perylene		0.14	1.02	11; 73%
	Phenathrene		27.2	103	14; 93%
	Pyrene		6.47	47.9	15; 100%
Polychlorinated biphenyls (PCBs)	3,3’-Dichlorobiphenyl	PCB 11	3.47	9.19	15; 100%
	2,4,4’-Trichlorobiphenyl	PCB 28	0.25	3.35	13; 87%
	2,2’,4,6’-Tetrachlorobiphenyl	PCB 47	0.13	0.22	3; 20%
	2,2’,4,6’-Tetrachlorobiphenyl	PCB 51	NA	NA	0
	2,2’,5,5’-Tetrachlorobiphenyl	PCB 52	0.13	2.89	9; 60%
	2,3’,4,5’-Tetrachlorobiphenyl	PCB 68	0.34	0.31	2; 13%
	2,2’,4,5,5’-Pentachlorobiphenyl	PCB 101	0.67	1.42	4; 27%
	2,3’,4,4’,5-Pentachlorobiphenyl	PCB 118	0.43	1.42	4; 27%
	2,2’,3,4,4’,5’-Hexachlorobiphenyl	PCB 138	0.54	0	1; 7%
	2,2’,4,4’,5,5’-Hexachlorobiphenyl	PCB 153	0.47	0.7	2; 13%
	2,2’,3,4,4’,5’-Hexachlorobiphenyl	PCB 180	0.92	0	1; 7%
	2,2’,3,4,4’,5’,6-Heptachlorobiphenyl	PCB 183	NA	NA	0
Pesticides	N,N-diethyl-meta-toluamide	DEET	138	5,160	14; 93%
	Atrazine		7.31	7.31	1; 7%
	Lindane		0.31	0.72	4; 27%
	Malathion		NA	NA	0
	Chlorpyrifos		2.76	2.76	1; 7%
	Cyprodinil		0.21	0.21	1; 7%
	Fipronil		10.8	4,110	11; 73%
	Trans-Chlordane		1.35	12.2	6; 40%
	Cis-Chlordane		5.27	8.22	3; 20%
	p,p’-Dichlorodiphenyldichloroethylene	DDE	0.36	5.61	9; 60%
	Chlorfenapyr		NA	NA	0
	Trifloxystrobin		2.8	4.54	3; 20%	
	Propiconazole		NA	NA	0
	Cis-Permethrin		13.1	21,400	15; 100%
	Trans-Permethrin		41.5	31,000	14; 93%
	Cypermethrin		15.6	92.1	3; 20%
	Pyraclostrobin		NA	NA	0
	Azoxystrobin		NA	NA	0
	Fluoxastrobin		NA	NA	0
Alkylphenols	4-tertoctylphenol	4tOP	14.2	41.3	15; 100%
	Nonylphenol isomer mix	NP	175	595	15; 100%
Phthalates	benzyl butyl phthalate	BBP	156	25,900	15; 100%
	dibutyl phthalate	DBP	362	11,500	15; 100%
	Bis (2-ethylhexyl) adipate	DEHA	1,130	2,730	8; 53%
	Bis(2-ethylhexyl) phthalate	DEHP	358	963	15; 100%
	Bis (2-ethylhexyl) terephthalate	DEHT	70,300	1,030,000	15; 100%
	di-ethyl phthalate	DEP	664	3,050	15; 100%
	di-isobutyl phthalate	DiBP	1,470	2,280	4; 27%
	di-isononyl phthalate	DINP	8,530	43,500	15; 100%
	di-methyl phthalate	DMP	154	199	3; 20%
	trioctylmetallitate	TOTM	368	7,120	15; 100%
Brominated flame retardants (BFRs)	2,4,4’-tribromodiphenyl ether	BDE 28	1.8	8.61	3; 20%
	2,2’,4,4’-tetrabromodiphenyl ether	BDE 47	13.2	605	14; 93%
	2,3’,4,4’-tetrabromodiphenyl ether	BDE 66	1.08	13.7	3; 20%
	2,2’,3,4,4’-pentabromodiphenyl ether	BDE 85	34.7	34.7	1; 7%
	2,2’,3,4,4’-pentabromodiphenyl ether	BDE 99	62.6	1,000	6; 40%
	2,2’,4,4’,6-pentabromodiphenyl ether	BDE 100	9.79	172	6; 40%
	2,2’,4,4’,5,5’-hexabromodiphenyl ether	BDE 153	83.8	83.8	1; 7%
	2,2’,4,4’,5,6’-hexabromodiphenyl ether	BDE 154	73.4	73.4	1; 7%
	2,2’,3,4,4’,5’,6-heptabromodiphenyl ether	BDE 183	1.67	1.67	1; 7%
	2-ethyl hexyl-2,3,4,5-tetrabromobenzoate	EHTBB	101	301	4; 27%
	Bistribromophenoxyethane	BTBPE	NA	NA	0
	Decabromodiphenyl ethane	DBDPE	2.07	4.35	4; 27%
	Decabromodiphenyl ether	BDE 209	7.37	13.6	8; 53%
	Octabromotrimethylphenyllindane	OBIND	NA	NA	0
	Tetrabromobisphenol A	TBBPA	NA	NA	0
	Tetrabutylphosphonium Hydroxide	TBPH	85	85	1; 7%
	Tris (2,3-dibromopropyl) isocyanurate	TDBPIC	NA	NA	0

The types and amounts of analytes detected varied substantially among participants. In total, 93 out of 119 chemicals (78%) were found in at least one participant household. Fifty-three chemicals (45% of the total number of chemicals) representing all eight chemical classes were detected in 50% (8 out of 15) of participant households (Table 4). These 53 chemicals represent all eight chemical classes.

**Table 4:** Class of chemicals and total number detected in at least 50% of households.

Chemical class	Total number of chemicals in class tested	Number of chemicals from class detected in at least 50% of households
Organophosphate Esters (OPE)	35	17
Brominated flame retardants (BFR)	7	2
Personal Care Product (PCP)	1	1
PAH	23	15
PCB	12	3
Pesticide	19	5
Alkylphenols (Phenol)	2	2
Phthalates	10	8

Twenty-five out of 119 (21%) individual chemicals were detected in all 15 dog tags (100%). The analytes detected in 100% of households included chemicals from the following classes: pesticides, organophosphates, phthalates, PAHs, alkylphenols, PCBs, and PCPs. They include cis-permethrin, di-ethyl phthalate, dibutyl phthalate, benzyl butyl phthalate, bis(2-ethylhexyl) phthalate, bis(2-ethylhexyl) terephthalate, di-isononyl phthalate, trioctylmetallitate, 4-tertoctylphenol, nonylphenol isomer mix, lilial, 3,3′-dichlorobiphenyl, fluoranthene, pyrene, benzo[j,b,k]fluoranthene, benzo[e]pyrene, benzo[a]pyrene, 2-isopropylphenyl diphenyl phosphate, 4-tert-butylphenyl diphenyl phosphate, tri-iso-butyl-phosphate, tris(chloroisopropyl) phosphate, bis(2-chloro-1-methylethyl)(2-chloropropyl) phosphate, triphenyl phosphate, 2-ethylhexyl diphenyl phosphate, and tris(2-ethylhexyl) phosphate.

## 4 Discussion

We employed a passive sampling method that has previously been shown to successfully assess environmental exposure in humans and their companion dogs (1, 5). We found that in a geographically diverse set of samples collected from dogs’ within the United States, several compounds representing eight chemical classes were detectable on silicone tags worn by dogs over a 120-h period. Finding sound methodology prompts further and broader investigation of chemical exposure in companion dogs via passive sampling.

Chemical exposure, broadly, has important implications for veterinary care and management. Many chemical toxicants found in and around the home environment (e.g., PBDEs, PCBs, phthalates) are the culprits in common companion animal diseases of the reproductive system, thyroid disorders, and various cancers in cats and dogs (14, 33). And, these conditions are among the top most frequently diagnosed (36). Improved understanding of where and how exposure occurs can inform care decisions when these patients present in the clinical setting.

Considering our results, the ubiquitous presence of several chemicals and compounds known to have adverse effects on human and animal health is especially concerning. However, additional data are needed to further assess what those specific concerns are or should be, and when we can and should act on them. With these data alone, we are limited in our ability to discern at what detectable level, frequency, and duration of exposure to one or several of the substances a dog’s health will be affected (or in what ways they will be impacted). Passive sampling techniques such as the silicone tag monitoring devices make it difficult to control for the effects of multiple routes and/or instances of exposure to various chemicals in isolation or in combination (though not likely any less so than more invasive physiological measures).

For example, in at least one case, an individual dog’s tag returned detectable amounts of several compounds at levels markedly higher than all other participants, including the presence of some chemicals not found in any other samples. In particular, the levels of the pesticides permethrin, fipronil, DEET and cypermethrin detected on this dog’s tag were the highest out of all samples. There are some studies that have shown that exposure to mixtures of these pesticides can have adverse effects (37, 38). In this case, the dog was reportedly in very good health according to the owner, with no new or ongoing health conditions at the time of sampling. The owner also reported using a flea and tick preventative that contained fipronil, so this result is consistent with a previous study showing statistically significantly higher levels of fipronil on dog tags worn by dogs treated with these types of flea and tick products (5).

The owner reported not using any pesticides in or around their home or property, but was notably one of only two participants who reported living in a rural area. DEET is a widely used insect repellent. While permethrin is a commonly used general use pesticide for residential and veterinary/medical applications, it is restricted-use for crops and wide areas (limits on application levels, except for as mosquito sprays) due to its aquatic toxicity, per the U.S. EPA. However, cypermethrin does have large scale commercial agricultural uses. We cannot determine if the exposure to the other pesticides represents an ongoing exposure in the immediate environment, or a brief exposure, such as an upwind pesticide application.

Due to the sample size of this initial deployment wherein our goal was to test logistical and methodological soundness, we were unable to investigate any potential direct correlation between risk of exposure and specific health effects and conditions in dogs. However, we do have access to medical records, diagnoses, ongoing care data, as well as household, physical, and social indicators for a potential sample size of up to more than 45,000 dogs. Our next deployment of silicone tags will support us in overcoming the limitations inherent in this pilot study and will be conducted in a way that takes full advantage of these additional data. With a larger sample, we will also be able to evaluate location-based co-factors without jeopardizing participants’ right to privacy, and with the express purpose of evaluating ambient environment data in conjunction with a comprehensive suite of additional variables.

Furthermore, the widespread adoption of low-burden, low-cost passive sampling tools and methods such as silicone monitoring devices should be more seriously considered as a means to increase equity and accessibility in data collection and to reach a more diverse participant demographic.

In feedback we received from 14 of the 15 study participants via a post-deployment survey, dog owners expressed that being a part of the data collection process was non-burdensome. All stated that the informational Zoom call was helpful, the instructions were clear and concise, and that the study activities were manageable. Two respondents reported difficulties with attaching the silicone tag to their dog’s collar, particularly with opening the attachment rings. One respondent reported difficulty with sending or receiving their package, but further details were not provided. When asked about the content they’d like to see in a results report, 10 respondents indicated a desire to receive their dog’s overall findings.

These considerations may be especially important for inclusion of populations of people and their dogs unjustly at higher risk of exposure and with lower access to care. And, because exposures can change considerably over time, seasonally, and in relation to various behaviors and activities, these tags offer an opportunity to repeatedly assess and compare risk, with relative ease.

While we recognize the limitations in attempting to assess multiple environmental exposures that can impact the health of dogs, studies using these passive sampling methods have great potential to support development of improved rapid response protocols. Given the successful detection of multiple exposures in a short period of time (120-h) during this study, silicone dog tags may offer a method for retrieving point-in-time risk exposure, such as during or immediately following an accident or disaster (39). In the past, risk assessment during such events has been only marginally effective, due in part to latency in data collection as well as using dated, non-location specific methods for assessment (40–42). Silicone tag monitoring devices, could enable a more accurate assessment of where and when exposure risk is highest, and to assess the capacity of community response and availability of resources to mitigate possible harm from exposures.

## 5 Conclusion

Data from this deployment of passive sampling devices indicate that silicone dog tags are an effective means to measure multiple chemical exposures in and around pet dogs’ homes. Given our results, veterinary practitioners should be cognizant of the potential hazards and adverse health outcomes for companion animals exposed to chemicals in the everyday environment. Our study also supports the value of using silicone tags with dogs to investigate potential health impacts on humans from shared environmental exposures. We will investigate this further in a future study that includes a hypothesis-generating deployment to a larger and geographically diverse sample of Dog Aging Project participants. We will deploy via a stratified selection process to recruit 80 additional dogs (20 small size/rural, 20 small size/urban, 20 large size/rural, 20 large size/urban) with a goal of further assessing location-based variation in exposures and potential compounding risk factors for both dogs and humans.

## Data availability statement

The datasets presented in this study can be found in online repositories. The names of the repository/repositories and accession number (s) can be found in the article/Supplementary material.

## Ethics statement

The animal studies were approved by Texas A&M University IACUC, under AUP 2021-0316 CAM. The studies were conducted in accordance with the local legislation and institutional requirements. Written informed consent was obtained from the owners for the participation of their animals in this study.

## Author contributions

RM: Investigation, Writing – original draft. CS: Writing – original draft. CW: Data curation, Formal analysis, Writing – review & editing. JO'B: Formal analysis, Validation, Visualization, Writing – review & editing. AK: Project administration, Writing – review & editing. MK: Data curation, Writing – review & editing. MD: Supervision, Writing – review & editing. HS: Conceptualization, Supervision, Writing – review & editing. AR: Conceptualization, Funding acquisition, Supervision, Writing – review & editing.

## DAP consortium

Joshua M. Akey; Brooke Benton; Elhanan Borenstein; Marta G. Castelhano; Amanda E. Coleman; Kate E. Creevy; Kyle Crowder; Matthew D. Dunbar; Virginia R. Fajt; Annette L. Fitzpatrick; Unity Jeffery; Erica C Jonlin; Matt Kaeberlein; Elinor K. Karlsson; Kathleen F. Kerr; Jonathan M. Levine; Jing Ma; Robyn L McClelland; Daniel E.L. Promislow; Audrey Ruple; Stephen M. Schwartz; Sandi Shrager; Noah Snyder-Mackler; M. Katherine Tolbert; Silvan R. Urfer; Benjamin S. Wilfond.
