# Supplementary material for: Silicone tags as an effective method of monitoring environmental contaminant exposures in a geographically diverse sample of dogs from the Dog Aging Project
Source: Front Vet Sci. 2024 Aug 16;11:1394061. doi: 10.3389/fvets.2024.1394061 (PMC11363705; doi:10.3389/fvets.2024.1394061)
Supplement: Supplementary file 1 [file Data_Sheet_1.pdf]

Supplementary Material

Silicone tags as an effective method of monitoring environmental contaminant exposures in a geographically diverse sample of dogs from the Dog Aging Project

Rylee Matheson[1†], Courtney L. Sexton[1†], Catherine F. Wise[2], Janice O'Brien[1], Amber J. Keyser[3], Mandy Kauffman[3], Matthew D. Dunbar[3], DAP Consortium, Heather M. Stapleton[2], Audrey Ruple[1*]

*** Correspondence:** Corresponding Author: aruple@vt.edu

1 Initial interest form

Silicone Tag Pilot Study Interest Form

Thanks for your interest in this Dog Aging Project Pilot Study! Please only complete this form if you are willing to:

1. Attend an introductory zoom call on **Saturday, 2/18, at 9 PST/12 EST** (or watch the recording in the following week).
2. Place a silicone dog tag on your dog's collar for 5 consecutive days between March 1 and March 15.
3. Send the silicone dog tag back to the Ruple Lab in a prepaid mailer.
4. Answer a short follow-up survey about your experience.

* Indicates required question

1. Your First Name *

2. Your Last Name *

3. Your Dog's Name *

If you have more than one dog, please enter the name of the dog who is a member of the Dog Aging Project Pack.

4. Email *

5. Phone *

Below please enter the mailing address where you want us to send the silicone tag.

Please note that the address you enter below will not update your address in your personal research portal.

6. Street Address *

7. City *

8. State *

9. Zip Code *

10. Please confirm that you are volunteering to participate in the Silicone Tag Pilot Study for the Dog Aging Project. *

If you have changed your mind and no longer want to participate, select NO.

Check all that apply.

Yes, I consent to participate in the Silicone Tag Pilot Study

No, I'm not interested at this time

Thank you for your willingness to participate in this pilot study!

Any questions about this pilot study can go directly to Dr. Ruple's Lab at ruplelab@gmail.com or via phone at 540-231-0342. IMPORTANT: Please add Dr. Ruple's email and phone to your address book so messages from her lab don't get marked as spam. Thanks!

Dog Aging Project

Longer, healthier lives. Together.

2 Participant instructions

SILICONE DOG TAG INSTRUCTIONS

If you have any questions, please email the Ruple Lab at ruplelab@gmail.com or call 540-231-0342.

The goal of this study is to use silicone dog tags to detect the environmental exposures that our dogs experience in their normal home environment. When worn on a dog's collar, the tags collect and absorb a wide variety of compounds that dogs might be exposed to from the air and surfaces.

HOW TO PARTICIPATE

- Use the key chain provided to attach the tag to your dog's collar.
- Try to minimize touching the silicone as much as possible while attaching the tag.
- **Leave collar and tag on your dog 24 hours a day for 5 consecutive days during the study period.** You can choose which 5 days, but they should be when you and your dog are having your normal routine at home (not on vacation or in another place).
- Once the tag is on your dog, **record the date and time of attachment** on the label of the bag.
- After 5 full days remove the tag from your dog's collar, wrap the tag tightly in the extra foil sheet provided, and place the wrapped tag back in the plastic bag.
- **Record the date and time tag was removed** on the label on the plastic bag.
- **Use the Notes section on the bag to record anything unusual** that you think we should know such as:
    - Application of flea, tick or heartworm prevention just before or during 5-day period
    - Extended visits to someplace other than your dog's normal home environment
    - Damage or loss of the tag (If the tag is lost, please email our lab for next steps)
    - Any unusual exposures not captured in the online Exposure Survey
- **Use the link in the email to complete the Exposure Survey as soon as you remove the tag.**
- Place the bag with the tag in the prepaid return mailer. **Put in any USPS mailbox as soon as possible.**
- If you cannot mail the tag back immediately, please store the tag in the freezer until you can.

Thank you for being part of this study!

3 Exposure survey

Dog Aging Project

Longer, healthier lives. Together.

EXPOSURE SURVEY

If you have any questions, please email the Ruple Lab at ruplelab@gmail.com or call 540-231-0342.

Your name:		Your dog's name:	

How long ago was your home built?	▢ <12 months ago<br>▢ 1-5 years ago<br>▢ 6-10 years ago<br>▢ 11-20 years ago<br>▢ 21-50 years ago<br>▢ > 50 years ago		How long has your dog lived in your current home?	▢ <6 months<br>▢ 6-11 months<br>▢ 1-3 years<br>▢ 4-8 years<br>▢ 9-12 years<br>▢ 12+ years
Which description best fits your home?	▢ Single family<br>▢ Condo/townhouse<br>▢ Apartment<br>▢ Multifamily<br>▢ Mobile home/RV<br>▢ Other		How many hours per day does your dog spend inside your home on average?	▢ 0-2 hours<br>▢ 3-5 hours<br>▢ 6-8 hours<br>▢ 9-12 hours<br>▢ 13-18 hours<br>▢ 19-22 hours<br>▢ 24 hours
Do you wear shoes in your home?	▢ Yes<br>▢ No		Does your dog have any in home personal care products such as perfumes or shampoos?	▢ Yes<br>▢ No

Is your dog exposed to passive tobacco smoke?	▢ Yes<br>▢ No		How often is your dog professionally groomed?	▢ Never<br>▢ 1x per year<br>▢ 2-6x per year<br>▢ 7-11x per year<br>▢ Monthly<br>▢ Weekly

During the 5-day period of this pilot study was your dog exposed to passive smoke from any source other than tobacco (e.g. wildfire, woodstove, vaping/e-cigarettes, candles)? Please list these exposures.

During the 5-day period of this pilot study, did your dog swim in any of the following water sources?

▢ Pond or lake
▢ River, stream or creek
▢ Swimming pool
▢ Ocean
▢ Agricultural ditch
▢ Other
▢ Did not swim

Please list all of the medications (other than flea, tick or heartworm) that your dog is currently taking.

Do you routinely apply flea or tick preventatives to your dog?

▢ Yes
▢ No

Did you apply a flea or tick preventative to your dog <u>during the 5 day study</u> period?	▢ Yes, topical ointment applied to skin<br>▢ Yes, currently wears a flea/tick collar<br>▢ Yes, oral medication<br>▢ No

Please list all the flea and tick medications given to your dog over the last year and their frequency.

Do you routinely give heartworm preventatives to your dog?	▢ Yes<br>▢ No
Did you give a heartworm preventative to your dog <u>during the 5 day study</u> period?	▢ Yes, topical ointment applied to skin<br>▢ Yes, oral medication<br>▢ No

Please list all the heartworm preventives given to your dog over the last year and their frequency.

Please list all the herbicides and pesticides used in your yard over the last year and their frequency (e.g. Roundup or 2,4-D). If applicable, please list lawn service used and frequency.

When is your dog permitted access to the lawn following herbicide and/or pesticide treatment:	▢ Immediately<br>▢ Less than 24 hours<br>▢ 24-48 hours<br>▢ Greater than 48 hours

Please list all the pesticides used INSIDE your home over the last year and their frequency (e.g. Raid or Ortho).

4 Participant feedback survey

Dog Aging Project Silicone Tag Pilot Study Feedback Survey

Thank you very much for being part of the Silicone Tag Pilot Study!

Our team is excited to learn from this pilot and develop the full study based on the data we've collected from the tags. Your feedback about the experience is also really important. Please help us understand what worked, what didn't, and how we could create a better experience by answering the questions below.

We really appreciate you and your dog!!

* Indicates required question

1. Your First Name *

2. Your Last Name *

3. Your Dog's Name *

4. Did you find the preparatory Zoom call helpful? *
Mark only one oval.

Yes

No

5. If no, please explain:

6. Were the instructions you received with the silicone tag clear and concise? *
Mark only one oval.

Yes

No

7. If no, please explain:

8. Did you have any difficulty attaching the silicone tag to your dog's collar? *

Mark only one oval.

Yes

No

9. If yes, please explain:

10. Did you have any difficulties receiving or sending your package? *
Mark only one oval.

Yes

No

11. If yes, please explain:

12. Do you think this activity is a manageable and reasonable request for the Dog Aging Project to make from participants? *

Mark only one oval.

Yes

No

13. Is there any information from our analysis of the silicone tags that you would be interested in receiving back in a "results report" of some kind?

14. Please share any additional comments or concerns you have regarding your experience.

Woof! We really appreciate your help and your feedback. You make our science possible, and you make it fun for us! Give your dog some extra love from our team!

Thank you again for your participation!

Dog Aging Project

Longer, healthier lives. Together.
